# Generalised weibull model-based approaches to detect non-constant hazard to signal adverse drug reactions in longitudinal data

**DOI:** 10.3389/fphar.2022.889088

**Published:** 2022-08-23

**Authors:** Odile Sauzet, Victoria Cornelius

**Affiliations:** ^1^ Department of Business Administration and Economics, Bielefeld University, Bielefeld, Germany; ^2^ Department of Epidemiology and International Public Health, Bielefeld School of Public Health (BiSPH), Bielefeld University, Bielefeld, Germany; ^3^ Imperial Clinical Trials Unit, School of Public Health, Imperial College London, London, United Kingdom

**Keywords:** pharmacoepidemiology, adverse events, signal detection, time-to-event models, electronic health records

## Abstract

Pharmacovigilance is the process of monitoring the emergence of harm from a medicine once it has been licensed and is in use. The aim is to identify new adverse drug reactions (ADRs) or changes in frequency of known ADRs. The last decade has seen increased interest for the use of electronic health records (EHRs) in pharmacovigilance. The causal mechanism of an ADR will often result in the occurrence being time dependent. We propose identifying signals for ADRs based on detecting a variation in hazard of an event using a time-to-event approach. Cornelius et al. proposed a method based on the Weibull Shape Parameter (WSP) and demonstrated this to have optimal performance for ADRs occurring shortly after taking treatment or delayed ADRs, and introduced censoring at varying time points to increase performance for intermediate ADRs. We now propose two new approaches which combined perform equally well across all time periods. The performance of this new approach is illustrated through an EHR Bisphosphonates dataset and a simulation study. One new approach is based on the power generalised Weibull distribution (pWSP) introduced by Bagdonavicius and Nikulin alongside an extended version of the WSP test, which includes one censored dataset resulting in improved detection across time period (dWSP). In the Bisphosphonates example, the pWSP and dWSP tests correctly signalled two known ADRs, and signal one adverse event for which no evidence of association with the drug exist. A combined test involving both pWSP and dWSP is reliable independently of the time of occurrence of ADRs.

## 1 Signal detection methods and longitudinal data

At the time a medicine is approved and receives market authorisation there should be robust evidence from clinical trials that the average benefit of therapy outweighs the average risk to the patient or at least to typical clinical trial participant. After a medicine has received licensed approval is released on the market, a much larger and more heterogeneous population will then receive the treatment. The aim of pharmacovigilance is to prevent the known harms and monitor the changes in frequencies to determine if the risk is higher than expected, and also to identify any new adverse drug reactions (ADRs). Pharmacovigilance is important for both newly released and established drugs and it can result in changes to prescribed labelling or, in extreme situations, to the medicine being withdrawn from the market. A key component of pharmacovigilance monitoring is the use of quantitative signal detection methods as a means to identify signals of ADRs that warrant further investigation. In this paper we aim to demonstrate the usability of novel model based approaches for quantitative signal detection.

Historically signal detection methods have been employed on data from spontaneous reporting systems (Hauben et al., 2005; Harpaz et al., 2012) with an aim to detect disproportionalities in frequencies between what is expected and what is observed (Bate et al., 1998; Evans et al., 2001; Szarfman et al., 2002; Van Puijenbroek Eugéne et al., 2002; Rothman et al., 2004; Noren et al., 2006). Over the last 15 years there has been increased interest in the use of electronic health records (EHRs) and longitudinal data to further enhance pharmacovigilance (Trifirò et al., 2011; Suling and Pigeot, 2012; Patadia et al., 2015). Longitudinal data available in EHR data differs from spontaneous reporting databases as it includes information on all patients who take the medicine (not just those that experience a suspected ADR), and all events that happen to that patient (not just those for which a potential causal association is suspected). It can also include detailed prescription issue information that allow treatment exposure and non-exposure to be estimated. Signal detection tests developed for spontaneous reporting systems, predominately rely on detecting a disproportionality in reporting between specific drug-adverse event combinations for the medicine of interest compared to all the other spontaneous reports for the other medicines combined. They do not utilise longitudinal information. These approaches rely on having access to a large and varied number of drugs so that a suitable “expected” value can be calculated from the combined medication reports. Longitudinal EHRs data offers considerable opportunities for improved pharmacovigilance over spontaneous reporting systems (Schuemie, 2011).

Recent signal detection methods have been proposed, which make optimal use of additional information available in longitudinal data. Methods include the modification of existing disproportionality approaches to incorporate person-time exposure into the Gamma-Poisson Shrinker test (Schuemie, 2011), identify patterns in the temporal association using the information component (Norén et al., 2010), use of time series combined with outlier analysis (Whalen et al., 2018), and change-point analysis (Trihn et al., 2018). Whilst these enhanced approaches make use of longitudinal information, they do not utilise the time of the adverse event occurrence at the individual level. We previously proposed a new approach that can be applied to single arm exposed drug cohorts (Cornelius et al., 2012) by developing a test which takes advantage of time-to-event information. The premise is based on recognising that the causal mechanism will often result in the occurrence of the ADRs being time dependent.

### Principle of a time-to-event approach

To take temporal processes into account, we considered using tests based on the hazard function of time-to-event. From here on, in order to aid clarity, we will refer to events not associated to the medication as adverse events (AEs). This definition differs slightly from the all-encompassing standard definition in which the event may or may not be associated. Events that are associated to the medication will be referred to as Adverse Drug Reactions (ADRs).

A hazard function represents the ‘instantaneous’ rate of occurrence of an event over time. It is always positive valued and when the hazard function is constant then this is considered to be consistent with observing a ‘background’ rate of events that are not associated with drug therapy (AEs). If the hazard function is non-constant this indicates a possible association of the event with initiation of drug therapy medication (ADRs). Previously we have developed a test based on the shape parameter for the Weibull distribution: the Weibull Shape Parameter test (WSP test) whose principle is to detect a variation in the hazard of an event over time (Cornelius et al., 2012). This methods has the advantage that it does not require a control group and it can be easily implemented using existing statistical software.

The WSP test has been shown to have good power under a range of scenarios with cohorts as small as 5,000 treated patients when the ADR occurred shortly after treatment start or at the end of the defined study period. However the data needed to be censored at various time point in order to reliably detect an ADR that occur in the middle of the observation period which increases the complexity of applying the test (Cornelius et al., 2012; Sauzet et al., 2013).

In this article, we propose to evaluate the reliability of two new tests, one based on a combination of the WSP test (dWSP) and one based on the power generalised Weibull distribution (pWSP). The power generalised Weibull distribution allow for more flexible hazard functions (Bagdonavičius and Nikulin, 2002). We show how the dWSP test as well as a test based on the power generalised Weibull distribution (pWSP) perform and complement each other through a simulation study. We apply the two tests to data from a cohort of women treated with biphosphonates obtained from the THIN database.

## 2 Models allowing for flexible hazard shape including a constant hazard

### 2.1 The weibull distribution based test

The simple version of this test (WSP) has been defined and its limitations discussed elsewhere (Cornelius et al., 2012; Sauzet et al., 2013). A Weibull distribution with a shape parameter equal to one is an exponential distribution with a constant hazard (the risk of AE ist constant over time). The main drawback of this model is that it can only model monotonous hazards and has therefore limited ability to detect signals when the increase in risk is in the middle of the observation period.

The hazard function

$$\lambda \left(t\right)=\alpha \theta^{\alpha }t^{\alpha -1}$$ (1)

Where *θ* is the scale parameter and *α* the shape parameter, is not time dependent when the latter is equal to one. The WSP (Weibull Shape Parameter) test consist of rising a signal if the 95% confidence interval for the estimated shape parameter *α* does not contain 1 (*α* is statistically different from 1).

### 2.2 The double weibull test

The WSP has proved to be of limited power for detecting a signal when an increase in risk occurs half way through the observation period due to symmetry of the hazard function. An improved test can therefore be achieved by breaking the symmetry through censoring the data at the middle of the observation period—thus reducing the observation period by half its duration—and performing the test twice: for the whole duration and for half of it. A signal is raised if one of the two shape parameters thus estimated is significantly different from 1. To control for multiple testing, we adjust the significance level to 0.025 for each individual test.

#### Detection across time period test

If the following null hypothesis for the shape parameters *α*_1_ (uncensored data) and *α*_0.5_ (censored data at mid-observation time) is rejected, a signal is raised:

$$H_{0}:\alpha_{1}=1\;and\;\alpha_{0.5}=1$$

and alternative hypothesis:

$$H_{1}:\alpha_{1}\neq 1\;or\;\alpha_{0.5}\neq 1$$

### 2.3 Power generalised weibull distribution based test

**FIGURE 1:**
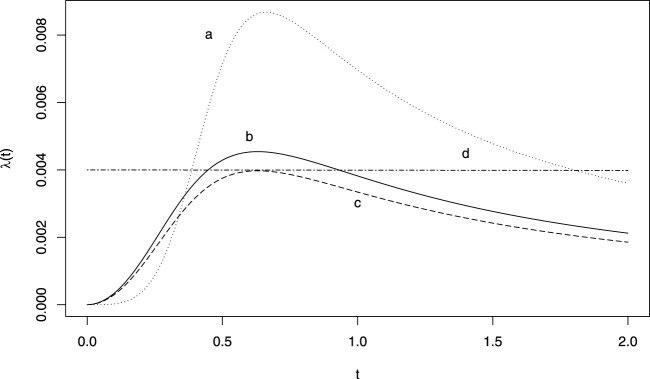
Example of hazard functions obtained from a generalised power Weibull distribution. **(A)** *ν* = 5, *γ* = 700 *θ* = 0.5; **(B)** *ν* = 3, *γ* = 700 *θ* = 0.5; **(C)** *ν* = 3, *γ* = 800 *θ* = 0.5; **(D)** *ν* = 1, *γ* = 1.5 *θ* = 167.

The power generalised Weibull distribution (PgW) introduced by Bagdonavičius and Nikulin (2002), is a generalisation of the Weibull distribution in which a second shape parameter allows for a wider range of forms of hazard functions. Some examples are provided in Figure 1. The advantage of this distribution for signal detection is that a unique set of shape parameters provide a constant hazard (exponential distribution), namely when they both equal one. We suggest the following pWSP test: if the two shapes parameters are significantly different from one both at a significance level of 0.05, then a signal is raised. Also when the parameters cannot be estimated (e.g., because of a non-convergence of the estimation algorithm), no signal is raised.

The survival function for the PgW with scale parameter *θ* and shape parameters (*ν*, *γ*) in the following:

$$S\left(t;\theta ,\nu ,\gamma \right)=\exp\left(1-{\left[1+{\left(\frac{t}{\theta }\right)}^{\nu }\right]}^{\frac{1}{\gamma }}\right)$$

with the following hazard function

$$\lambda \left(t\right)=\frac{\nu }{\gamma \theta^{\nu }}t^{\nu -1}{\left[1+{\left(\frac{t}{\theta }\right)}^{\nu }\right]}^{\frac{1}{\nu }-1}$$

#### 2.3.1 Power generalised weibull distribution test

If the following null hypothesis for the shapes parameters *ν* and *α* is rejected with a significance level of 0.05, then a signal is raised:

$$H_{0}:\nu =1\;or\;\gamma =1$$

with the alternative hypothesis:

$$H_{1}:\nu \neq 1\;and\;\gamma \neq 1$$

### 2.4 Estimation

The parameters of the power Weibull model can be estimated using a numerical maximisation of the likelihood (MacDonald, 2014) which can be easily implemented in *R*. The confidence intervals for the shape parameters are obtained from the Hessian matrix and no bootstrapping method is necessary. However the number of events to be expected in signal detection can be low and the convergence of the numerical estimation is not guaranteed.

We adapted the numerical maximisation of the likelihood for a mixture of Poisson distribution presented by MacDonald (MacDonald, 2014) to the case of a power generalised Weibull distribution for censored data. The likelihood function for censored data with density function *f* and survival function *S*(*t*) = 1 − *P* (*T* > *t*) is given in closed form for the power Weibull distribution by:

$$L\left(\theta \right)=\overset{n}{\underset{i=1}{\prod }}f{\left(t_{i},\theta \right)}^{\delta_{i}}S{\left(t_{i},\theta \right)}^{1-\delta_{i}},$$

Where *θ* is the vector of parameters to be estimated, and the data consists of *n* observation ${\left(t_{i},\delta_{i}\right)}_{i\in 1,\ldots ,n}$ , *t*_*i*_ being the time at which either the adverse events occurs or the time at which the observation has been censored and *δ*_*i*_ is a censoring indicator which equal one if an event is observed and 0 if not.

Confidence intervals are obtained by inverting the Hessian matrix (Millar, 2011) if it is non-singular. In case the nlm algorithm fails to converge or of a singular Hessian matrix, the test results are that no signal is raised.

The R code for the estimation of the PgW distribution is provided in the Supplementary Material.

## 3 Simulation study

In order to evaluate the performance of dWSP and pWSP, background adverse event data (AEs) were simulated using an exponential distribution. The simulated background rates were 1 (uncommon event), 5, and 10% (common) over the period of observation. The rate of drug related events (ADRs) were of 10, 20, 50 and 100% of background rates. While this approach differs to the common denomination of rare (1/10 000), uncommon (1/100), or common (1/10) events rates, this is the relevant approach in term of signal detection of ADRs within background events.

The time at which ADRs occurred was simulated using a normal distribution with varying standard deviations to reflect different variability in the report of events. The mean date was either the middle point of the observation period defined by the censoring date, the first quarter, or the third quarter. Negative dates were removed from the analysis. We chose not to use parametric or semi-parametric time-to-event models for the simulation of events so as not to obtain too similar distributions to the models used for testing. A normal distribution provides a reasonable representation of how events would occur in practice if due to an ADR that was time dependent.

For each set of simulation parameters 1,000 datasets of background events (AEs) and drug related event (ADRs) were simulated. The models were fitted to the data with drug related events and without to compare rates of false positive and rate of false negative.

Rates of true (*TP*) and false positives (*FP*) were obtained as a measure of sensitivity and (1-)specificity and results were also presented in terms of accuracy:

$$acc=\frac{TP+1-FP}{2}=\frac{Sensitivitiy\;+\;Specificity}{2}$$

The simulations were performed with R (R Core Team, 2020) using the package survival (Therneau, 2015).

## 4 Results

Results are presented for scenarios which produced an average of at least 11 ADRs and no more than 300 to cover only realistic scenarios.

The average sample sizes obtained are given in Table 1 for an observed population of 2,500. For populations of 5,000 and 10 000, the number can be multiplied accordingly.

**TABLE 1:** Average sample sizes for an simulated population of 2,500. ADR: adverse drugs reaction.

Background rate									
(AEs) (%)	5	10	5	10	1	5	10	1	5
Rate of ADRs									
(% of backg. rate)	10	10	20	20	50	50	50	100	100
Observations									
with no events	2,378	2,262	2,378	2,262	2,475	2,378	2,262	2,474	2,377
Background									
events (AEs)	122	238	122	238	25	122	238	25	122
ADRs	13	27	27	54	13	68	136	24	122

### 4.1 Effect of the standard deviation of reported adverse drug reactions

We have simulated three standard deviations for the reporting of ADRs to check how the variability in the reporting time of ADRs would affect the rate of false and true positives.

According the average values given in Table 2, there is no indication that the accuracy is affected by the standard deviation to the exception of the dWSP test for ADRs occurring at the middle of the observation period. In that case the accuracy seems to be decreasing slightly with increasing standard deviation.

**TABLE 2:** Average accuracy over all scenarios for pWSP and dWSP tests for varying standard devastations (SD) of ADR reporting.

	1st quarter		Middle		3rd quarter	
SD	pWSP	dWSP	pWSP	dWSP	pWSP	dWSP
0.05	0.81	0.67	0.74	0.75	0.51	0.75
0.1	0.81	0.67	0.75	0.75	0.51	0.75
0.5	0.80	0.67	0.74	0.73	0.51	0.75

### 4.2 Relative performance of the power generalised weibull distribution and the detection across time period

#### 4.2.1 False positives

**FIGURE 2:**
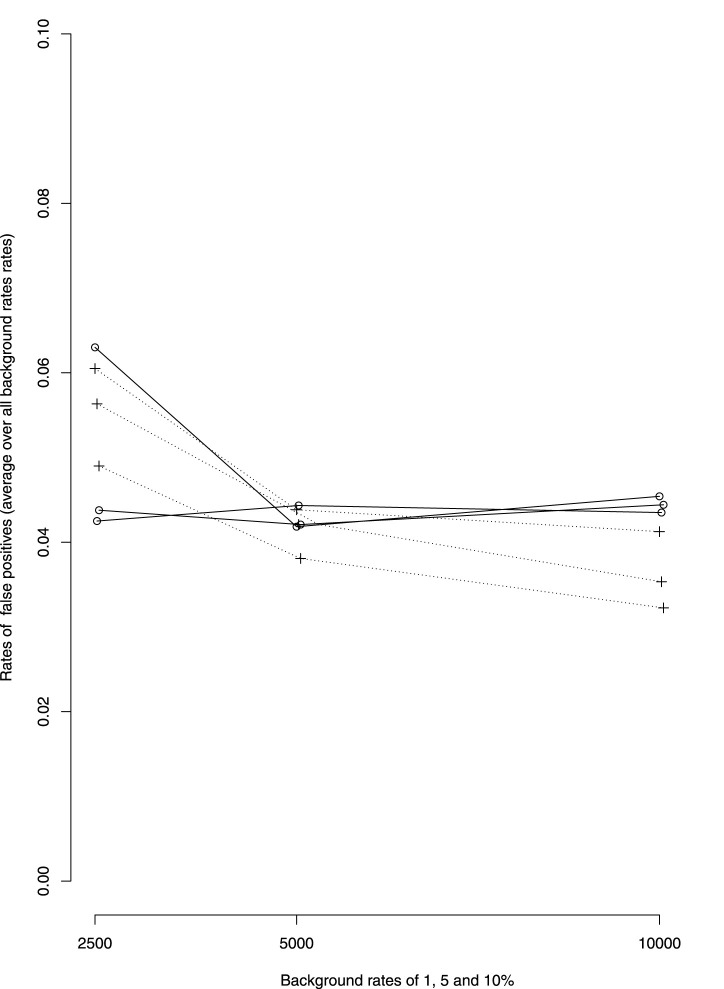
Average over all rates of ADRs of false positive rates by background rates (1, 5 and 10%, rates increasing with background rates). —dWSP …pWSP.

There seem to be little differences in the rate of false positive between dWSP and pWSP (Figure 2). On average these rates remain below 6% for the smaller sample sizes which is close the 5% significance level. The rate of false positive decreases with increasing sample size for the pWSP to reach 4% whereas it seems to remain on average over all rates of ADRs or all background rates at around 5%.

#### 4.2.2 First quarter of the observation period

**FIGURE 3:**
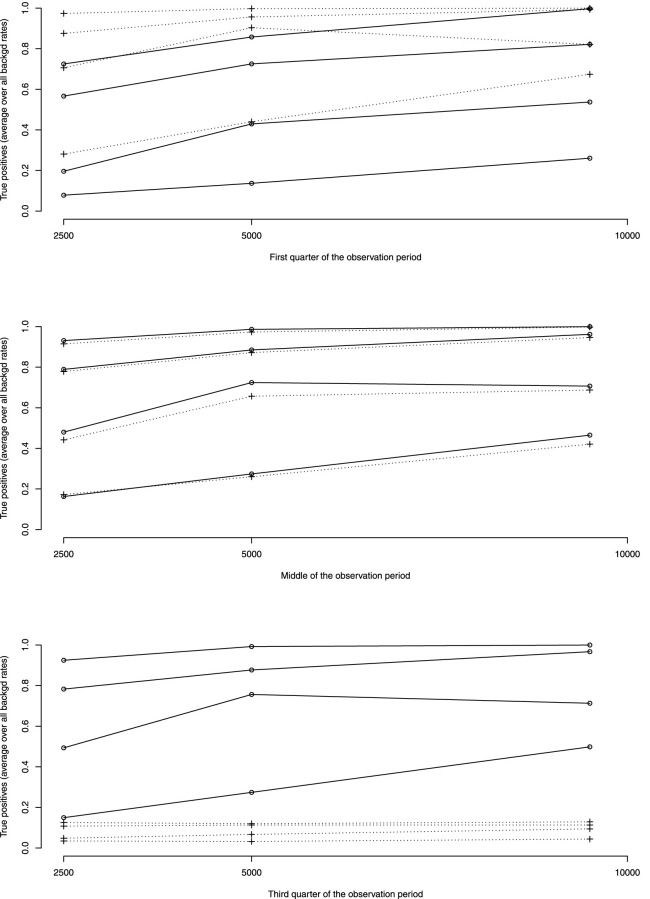
Average over all background rates of true positive rates by rates of ADRs as percentage of background rates (10, 20, 50 and 100%, rates increasing with ADRs rates). —dWSP …pWSP.

Figure 3 shows the rates of true positive for ADRs simulated occurring in the first quarter of the observation period per rate of ADRs on average taken over all other simulation parameters. Table 3 shows the average rates of true positives per effectively simulated numbers of ADRs. For ADRs occurring during the first quarter of the observation period the pWSP test ist very effective for a relatively small number of observed ARDs with an average of true positive rate reaching 80% from about 50 observed ADRs. About 100 observed ADRs are necessary for dWSP to reach a true positive rate of 80%. For all number of ADRs the pWSP performs better than dWSP. For example a rate of 79% is reached by pWSP for about 50 observed ADRs whereas the rate is 32% for dWSP.

**TABLE 3:** Mean true positive rates per simulated number of ADRs and time of occurence.

Nb of ADRs	< 20	20–30	45–55	68	98–136	> 200
1st quarter of observation period						
pWSP	0.42	0.59	0.79	0.99	0.97	1.00
dWSP	0.06	0.13	0.32	0.64	0.78	1.00
Middle of observation period						
pWSP	0.29	0.40	0.58	0.91	0.87	0.99
dWSP	0.27	0.41	0.61	0.95	0.91	1.00
3rd quarter of observation period						
pWSP	0.08	0.07	0.05	0.09	0.10	0.14
dWSP	0.24	0.40	0.63	0.98	0.92	1.00

**FIGURE 4:**
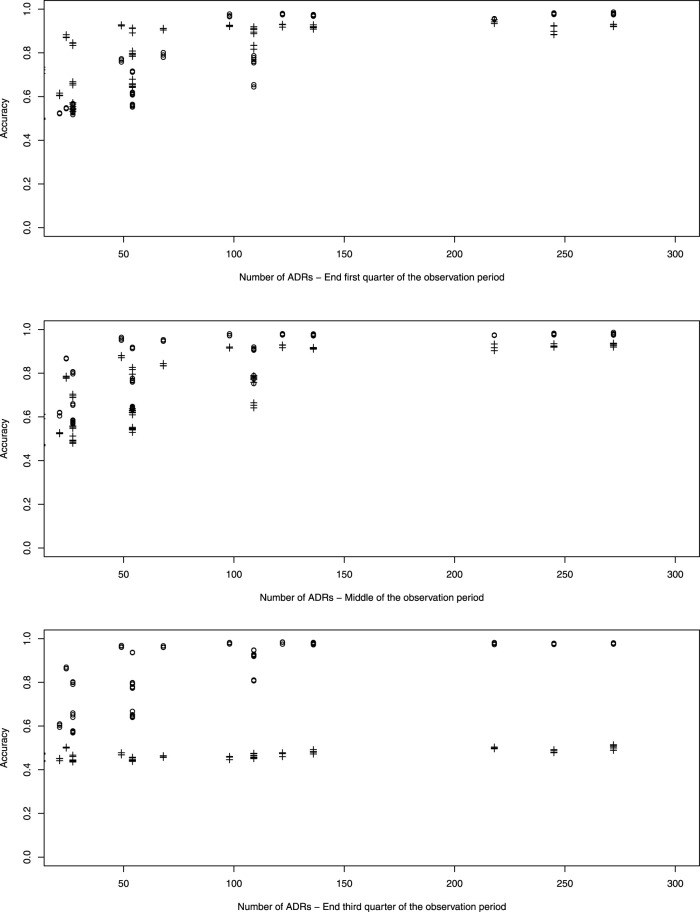
Plot of accuracy against the number of simulated ADRs. dWSP: +; pWSP: ◦

Figure 4 which represents the accuracy against the number of ADRs in more details, shows that for ADRs occurring at the first quarter of observation time the performance of the pWSP test is an improvement over the one of the dWSP by showing good performance also for small number of ADRs (see also below). Figuretp shows that for the pWSP test, the rate of true positives reaches above 80% for an increase of 50% of more of the background rate for a number of observation of 2,500.

#### 4.2.3 Middle of the observation period

For ADRs simulated at the middle of the observation period the dWSP tests performs slightly better than the pWSP test according to averages shown in Figure 4. For the highest average a rate of accuracy or true positive of above 90% is reached. Table 3 shows that the rate of true positive for both pWSP dWSP is slightly lower than the rates obtained by pWSP in the first quarter of observation for about 50 observed ADRs. However by 68 observed ADRs an true positive rate of 95% is achieved by dWSP which still makes it a reliable test for a relatively small number of ADRs. Figuretp shows that for both the pWSP and the dWSP tests, the rate of true positives reaches above 80% for an increase of 50% or more of the background rate for a number of observation of 2,500.

#### 4.2.4 Third quarter of the observation period

Here the comparison shows that the pWSP is not suitable to flag ADRs which occurs toward the end of the simulation period (Figure 3 and Table 3). On the other end the dWSP gives suitably good results with averages accuracy and true positive rates reaching well above 90% from 68 observed ADRs. The performance of dWSP is slightly better that at the middle of the observation period for a number of observed ADRs of 68 or more. For smaller number of ADRs it performs less well. Figuretp shows that for the dWSP test, the rate of true positives reaches above 80% for an increase of 50% or more of the background rate for a number of observation of 2,500.

#### 4.2.5 Small sample sizes of adverse drug reactions

The performance of pWSP and dWSP for small sample sizes of ADRs depends strongly on the background rates. If the events are rare in the population then ADRs are easier to detect. In particular in the first quarter of the observation period the pWSP test detected an average of 78% of true positive for sample sizes of ADRs of 50 or less for background rates of 1% whereas this number reduces to 37% for background rates of 5%. For the middle and the third quarters of the observation period the maximal rate of true positive for background rates of 1% in about 60%.

#### 4.2.6 Complementarity of power generalised Weibull distribution and detection across time period

The simulations indicate that when there are 120 ADRs or more, the pWSP or dWSP can provide a test accuracy of 100% independently of the background rate. But as Figure 4 shows, for smaller sample sizes the background rate plays a role on how well the tests performs overall.

Moreover we see a complementarity of the two tests in which the pWSP detect better ADRs occurring in the first half of the observation period and the dWSP in the second half. Very small numbers of observed ADRs are better detected when they occur in the first half of the observation period than later.

## 5 Example

The Health Improvement Network (THIN) is a database containing the electronic healthcare records of over 550 United Kingdom general practices covering almost 6% of the UK population. The records started in 2002 and provide longitudinal prescribing and reported event data for each patient of the participating practices (Trifirò et al., 2011).

The dataset used in this study consists of female patients in the THIN dataset, who have been prescribed bisphosphonates for the treatment of osteoporosis to the exclusion of patient with a history of malignant cancer or Paget’s disease (prescription included one of the following drug: alendronate, etidronate, ibandronic acid, palmidronate, zolidronic acid, risedronate, tiludronate and clodronate). The index date for each patient is the date of first prescription of a bisphosphonate. Unless the patient died or left the practice, all observations ended in August 2008. There were 19,817 prescribed women in the data. Any event recorded after the index date (date of first prescription) is included in the analysis. The end date was defined by the earliest of transfer out of the practice date, death date, or last data collection date. A detailed analysis of the data using the WSP tool can be found in Sauzet et al. (2013).

Here we applied the two tests dWSP and pWSP with the same events with varying incidence rates and reporting pattern by patients: headache, musculoskeletal pain, alopecia and carpal tunnel syndrome (CTS). Headache and musculoskeletal pain events are known to be associated to bisphosphonates whereas evidence of association for alopecia and carpal tunnel syndrome is lacking. Due to limited means the number of AE investigated had to remain small and the number of cases available had to be sufficient within the observation time, which excluded well knows ADRs like osteonecrosis of the jaw. For headache the observation time was 15 days after starting the medication, for musculoskeltal pain the observation period was 90 days from starting the medication whereas this period was of 1 year for alopecia and CTS.

The two tests provided consistent results: both correctly identified headache and pain as potential ADRs, and for the AEs with unknown association, they did not raise a signal for carpal tunnel, but did raised a signal for alopecia being possibly associated with starting treatment. Despite the small number of cases of headache the estimation algorithm for pWSP did converge. Results are provided in Table 4.

**TABLE 4:** Signal raised from the pWST and dWSP to a cohort of 19,817 women prescribed with biphosphonates (THIN dataset). ∗Mean simulation values based on an occurrence around the first quarter of the observation period and based on the number of observations. TP, True positive rate (sensitivtiy); FP, False positive rate (1- specificity).

Outcome	Cases	Estimated accuracy∗	Observation	pWSP	dWSP	Published Evidence
Headache	12	5% FP + 42% TP	15 days	signal	signal	Association
Muscoskeletal pain	104	5% FP + 97% TP	90 days	signal	signal	Association
Carpal Tunnel	96	5% FP + 97% TP	365 days	no signal	no signal	No evidence
Alopecia	76	5% FP + 99% TP	365 days	signal	signal	No evidence

## 6 Conclusion

We have compared the ability of two complementary distributional approaches for time-to-event models to raise signals for adverse drug reactions by testing if a hazard was (almost) constant. The aim was to a detect potential relationship between a drug and an adverse event without at this stage aiming at establishing a causal relationship. Because the Weibull distribution can only model increasing or decreasing hazard functions, previous work has shown the limitation of a test based on the Weibull distribution if the increased hazard occurred at the middle of the observation period. In this work we compared the usability of an approach based on the power Weibull distribution. This distribution offers more flexibility for the shape of the hazard function including a constant hazard (pWSP). We also presented an improved Weibull based test applied on the whole of the observation period and on data censored at mid-observation (dWSP). The two test complement each other by performing well for different periods of the observation time.

Censoring data at the middle of the observation period improve the performance of the WSP test used alone for ADRs occurring at the middle of the observation period without outperforming the pWSP for ADRs occurring in the first half of the observation period. The pWSP did provide satisfying results, performing also better than the dWSP, for ADRs occurring in the first quarter of the observation period. However the pWSP failed to detect increased hazard occurring toward the end of the observation period. By using a range of simulation scenario including varied sample sizes we have seen that a combination of these models would provide a accuracy above 80% event for samplean number of observation as small as 2,500 depending on background rates.

Our recommendation in view of the simulation results would be to use a test based on a combination of dWSP and pWSP. Future work should establish what are the minimal sample size relative to background rates for a reliable signal detection for a dual pWSP-dWSP test. Optimal significance level should also be obtained. However it is now clear that these test should be implemented in the routine investigations to flag potential associations between adverse events and medicines using electronic health records data.

We acknowledge the financial support of the German Research Foundation (DFG) and the Open Access Publication Fund of Bielefeld University for the article processing charge.

## Data Availability

Publicly available datasets were analyzed in this study. This data can be found here: https://www.the-health-improvement-network.com/.

## References

[B1] Bagdonavičius V. Nikulin M. S. (2002). Accelerated life models. of Monographs on statistics and applied probability. Boca Raton, Calif: Chapman & Hall/CRC, 94.

[B2] Bate A. Lindquist M. Edwards I. R. Olsson S. Orre R. Lansner A. (1998). A Bayesian neural network method for adverse drug reaction signal generation. Eur. J. Clin. Pharmacol. 54 (4), 315–321. 10.1007/s002280050466 9696956

[B3] Cornelius V. R. Sauzet O. Evans S. J. (2012). A signal detection method to detect adverse drug reactions using a parametric time-to-event model in simulated cohort data. Drug Saf. 35 (7), 599–610. 10.2165/11599740-000000000-00000 22702641

[B4] Evans S. J. Waller P. C. Davis S. (2001). Use of proportional reporting ratios (PRRs) for signal generation from spontaneous adverse drug reaction reports. Pharmacoepidemiol. Drug Saf. 10 (6), 483–486. 10.1002/pds.677 11828828

[B5] Harpaz R. DuMouchel W. Shah N. H. Madigan D. Ryan P. Friedman C. (2012). Novel data-mining methodologies for adverse drug event discovery and analysis. Clin. Pharmacol. Ther. 91 (6), 1010–1021. 10.1038/clpt.2012.50 22549283 PMC3675775

[B6] Hauben M. Madigan D. Gerrits C. M. Walsh L. Van Puijenbroek E. P. (2005). The role of data mining in pharmacovigilance. Expert Opin. Drug Saf. 4 (5), 929–948. 10.1517/14740338.4.5.929 16111454

[B7] MacDonald I. L. (2014). Numerical maximisation of likelihood: A neglected alternative to em? Int. Stat. Rev. 82 (2), 296–308. 10.1111/insr.12041

[B8] Millar R. B. (2011). SAS and ADMB maximum likelihood estimation and inference: With examples in R, 111. John Wiley & Sons.

[B9] Noren G. N. Bate A. Orre R. Edwards I. R. (2006). Extending the methods used to screen the WHO drug safety database towards analysis of complex associations and improved accuracy for rare events. Stat. Med. 25 (21), 3740–3757. 10.1002/sim.2473 16381072

[B10] Norén G. N. Hopstadius J. Bate A. Star K. Edwards I. R. (2010). Temporal pattern discovery in longitudinal electronic patient records. Data Min. Knowl. Discov. 20, 361–387. 10.1007/s10618-009-0152-3

[B11] Patadia V. K. Coloma P. Schuemie M. J. Herings R. Gini R. Mazzaglia G. (2015). Using real-world healthcare data for pharmacovigilance signal detection–the experience of the EU-ADR project. Expert Rev. Clin. Pharmacol. 8 (1), 95–102. 10.1586/17512433.2015.992878 25487079

[B12] R Core Team (2020). R: A language and environment for statistical computing. Vienna, Austria: R Foundation for Statistical Computing. Availableat: https://www.R-project.org .

[B13] Rothman K. J. Lanes S. Sacks S. T. (2004). The reporting odds ratio and its advantages over the proportional reporting ratio. Pharmacoepidemiol. Drug Saf. 13 (8), 519–523. 10.1002/pds.1001 15317031

[B14] Sauzet O. Carvajal A. Escudero A. Molokhia M. Cornelius V. R. (2013). Illustration of the weibull shape parameter signal detection tool using electronic healthcare record data. Drug Saf. 36 (10), 995–1006. 10.1007/s40264-013-0061-7 23673816

[B15] Schuemie M. J. (2011). Methods for drug safety signal detection in longitudinal observational databases: LGPS and LEOPARD. Pharmacoepidemiol. Drug Saf. 20 (3), 292–299. 10.1002/pds.2051 20945505

[B16] Suling M. P. I. Pigeot I. (2012). Signal detection and monitoring based on longitudinal healthcare data. Pharmaceutics 4 (4), 607–640. 10.3390/pharmaceutics4040607 24300373 PMC3834930

[B17] Szarfman A. Machado S. G. O’Neill R. T. (2002). Use of screening algorithms and computer systems to efficiently signal higher-than-expected combinations of drugs and events in the US FDA's spontaneous reports database. Drug Saf. 25 (6), 381–392. 10.2165/00002018-200225060-00001 12071774

[B18] Therneau T. M. (2015). A package for survival analysis in S.

[B19] Trifirò G. Patadia V. Schuemie M. J. Coloma P. M. Gini R. Herings R. (2011). EU-ADR healthcare database network vs. spontaneous reporting system database: Preliminary comparison of signal detection. Stud. Health Technol. Inf. 166, 25–30. 21685607

[B20] Trihn N. T. H. Sole E. Benkebil M. (2018). Benefits of combining change-point analysis with disproportionality analysis in pharmacovigilance signal detection. Pharmacoepidemiol Drug Saf. 6, 1–7. 10.1002/pds.4613 29992679

[B21] Van Puijenbroek Eugéne P. Andrew B. Marie L. Roland O. (2002). A comparison of measures of disproportionality for signal detection in spontaneous reporting systems for adverse drug reactions. Pharmacoepidemiol. Drug Saf. 11 (1), 3–10. 10.1002/pds.668 11998548

[B22] Whalen E. Hauben M. Bate A. (2018). Time series disturbance detection for hypothesis-free signal detection in longitudinal observational databases. Drug Saf. 41 (6), 565–577. 10.1007/s40264-018-0640-8 29468602

