# Supplementary material for: Generalised weibull model-based approaches to detect non-constant hazard to signal adverse drug reactions in longitudinal data
Source: Front Pharmacol. 2022 Aug 23;13:889088. doi: 10.3389/fphar.2022.889088 (PMC9445551; doi:10.3389/fphar.2022.889088)
Supplement: Supplementary file 1 [file DataSheet1.PDF]

1 Hypotheses für signal detection tests

WSP test: A Weibull model is fitted to the time-to-event data. If the following null hypothesis for the shape parameter α is rejected, a signal is raised.

$$H_0 : \alpha = 1$$

and alternative hypothesis:

$$H_1 : \alpha \neq 1$$

The double Weibull shape parameter test, which will be assessed in this work, can be described as follow:

dWSP test: If the following null hypothesis for the shape parameters α_1 (uncensored data) and $\alpha_{0.5}$ (censored data at mid-observation time) is rejected, a signal is raised:

$$H_0 : \alpha_1 = 1 \textit{ and } \alpha_{0.5} = 1$$

and alternative hypothesis:

$$H_1 : \alpha_1 \neq 1 \textit{ or } \alpha_{0.5} \neq 1$$

pWSP test: If the following null hypothesis for the shape parameters ν and γ is rejected, a signal is raised:

$$H_0 : \nu = 1 \textit{ or } \gamma = 1$$

with the alternative hypothesis:

$$H_1 : \nu \neq 1 \textit{ and } \gamma \neq 1$$

2 Examples of power Weibull distributed hazard functions

See Figure 1

3 Estimation

The parameters of the power Weibull model can be estimated using a numerical maximisation of the likelihood [1] which can be easily implemented in R. This has the advantage over the EM algorithm that the confidence intervals for the shape parameters can be obtained from the Hessian matrix and no bootstrapping method is necessary. However the number of events to be expected in signal detection can be low and the convergence of the numerical estimation is not guaranteed.

We adapted the numerical maximisation of the likelihood for a mixture of Poisson distribution presented by MacDonnald [1] to the case of a power generalised Weibull distribution for censored data. The likelihood function for censored data with density function f and survival function $S(t) = 1 - P(T > t)$ is given in closed form for the power Weibull distribution by:

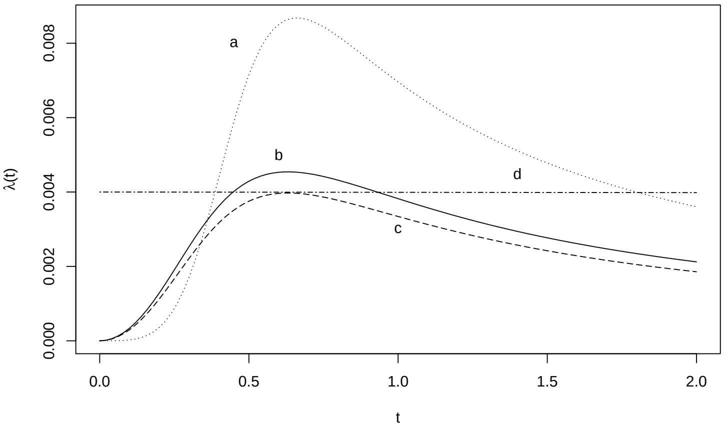

Figure 1: Example of hazard functions obtained from a generalised power Weibull distribution. a. $\nu = 5,\ \gamma = 700\ \ \theta = 0.5$; b. $\nu = 3,\ \gamma = 700\ \ \theta = 0.5$; c. $\nu = 3,\ \gamma = 800\ \ \theta = 0.5$; d. $\nu = 1,\ \gamma = 1.5\ \ \theta = 167$

$$L(\theta) = \prod_{i=1}^{n} f(t_i, \theta)^{\delta_i} S(t_i, \theta)^{1-\delta_i},$$

where θ is the vector of parameters to be estimated, and the data consists of n observation $(t_i, \delta_i)_{i\in 1,\ldots,n}$, t_i being the time at which either the adverse events occurs or the time at which the observation has been censored and δ_i is a censoring indicator which equal one if an event is observed and 0 if not.

The *R* code using the package *survival* [2] for the density and survival functions and the function *nlm* for the maximisation is provided in the appendix.

Confidence intervals are obtained by inverting the Hessian matrix [3] if it is non-singular. In case the nlm algorithm fails to converge or of a singular Hessian matrix, the test results are that no signal is raised.

4 R Code for the pWSP test

The code for the pWSP is provided below:

Likelihood function:

```
pWSPlikelihood=function(param,x)
{ theta<-exp(param[3])
  lambda=exp(param[1:2])
    dersurv<-function(x)((lambda[2-1]/(lambda[3-1]*theta^lambda[2-1]))
```

```
*x[1]^(lambda[2-1]-1)*
(1+(x[1]/theta)^lambda[2-1])^(1/lambda[3-1]-1)
*exp(1-(1+(x[1]/theta)^lambda[2-1])^(1/lambda[3-1])))^x[2]
*(exp(1-(1+(x[1]/theta)^lambda[2-1])^(1/lambda[3-1])))^(1-x[2])
   foo=-sum(log(apply(x,1,dersurv)))
  foo
}

start<-c(0,0,0)
res<-try(nlm(pWSPlikelihood,start,Data,hessian=T))
   if(is.vector(res)==F)
     {respWSP=NA
    }
   else{
  vhat<-try(solve(res$hessian))
if(is.matrix(vhat)==F)
{test.pWSP=NA
}
  else{
 test.pWSP<-(prod(sign(exp(res$estimate[1]+c(-1,1)*1.96*sqrt(vhat[1,1]))-1))
+1+prod(sign(exp(res$estimate[2]+c(-1,1)*1.96*sqrt(vhat[2,2]))-1))+1)/2
  } }
```

5 Details of simulation method

Varying background rates were obtained by using different censoring times (censoring: 0.1,0.5,1) which provided background rates of 1 (uncommon event), 5 and 10% (common). The rate of drug related events (ADRs) was defined as a percentage of the theoretical background rate (0.27, 0.15, 0.06, 0.03) $* \, censoring$ corresponding to rates of ADRs of 10, 20, 50 and 100% of background rates. While this approach differs to the common denomination of rare (1/10 000), uncommon (1/100), or common (1/10) events rates, this is the relevant approach in term of signal detection of ADRs within background events.

The time at which ADRs occurred was simulated using a normal distribution with varying standard deviations to reflect different variability in the report of events (0.005, 0.01, 0.05)$*censoring$. The mean date was either the middle point of the observation period defined by the censoring date, the first quarter, or the third quarter. Negative dates were removed from the analysis. We chose not to use parametric or semi-parametric time-to-event models for the simulation of events so as not to obtain too similar distributions to the models used for testing. A normal distribution provides a reasonable representation of how events would occur in practice if due to an ADR that was time dependent.

References

1 MacDonald IL. Numerical maximisation of likelihood: A neglected alternative to EM? International Statistical Review. 2014;82(2):296–308.

2 Therneau TM. A Package for Survival Analysis in S; 2015.

3 Millar RB. Maximum likelihood estimation and inference: with examples in R, SAS and ADMB. vol. 111. John Wiley & Sons; 2011.
